# An Indonesian female with pulmonary cystic hamartoma: a case report and literature review

**DOI:** 10.1097/MS9.0000000000000137

**Published:** 2023-02-06

**Authors:** Gemilang Khusnurrokhman, Anna Febriani, Priangga A. Wiratama, Anita Widyoningroem

**Affiliations:** a Department of Pulmonology and Respiratory Medicine; b Department of Anatomical Pathology; c Department of Radiology, Faculty of Medicine, Universitas Airlangga – Dr. Soetomo General Academic Hospital, Surabaya, Indonesia

**Keywords:** pain, pulmonary cystic hamartoma, oncology, wedge resection

## Abstract

**Introduction:**

Pulmonary cystic hamartoma is a rare benign cystic mass of the lung with clinical symptoms and radiological features that are not typical.

**Case presentation:**

A 43-year-old Indonesian female complained of chest and right shoulder pain, especially in the right clavicle. The patient underwent a chest X-ray and computed tomography scan thorax contrast, resulting in an anterior mediastinal tumor. The patient underwent wedge resection, and anatomical pathology showed pulmonary cystic hamartoma. The patient experienced postsurgery improvement.

**Discussion:**

Pulmonary cystic hamartoma does not have typical signs and symptoms. Pulmonary hamartoma diagnosis cannot be confirmed until a pathology anatomy examination is performed. Wedge resection is the first choice to treat pulmonary cystic hamartoma.

**Conclusion:**

Pulmonary cystic hamartoma is diagnosed with examination from pathology anatomy.

HIGHLIGHTS

- Preoperative diagnosis and treatment are difficult to establish in pulmonary cystic hamartoma.
- Wedge resection is the first choice for the management of pulmonary cystic hamartoma.
- Pulmonary cystic hamartoma is a rare benign cystic mass of the lung.

## Introduction

Pulmonary cystic hamartoma is a rare benign cystic mass of the lung with no specific clinical or radiological signs. Pathological examination typically presents cysts that have grown slowly^1^. It is present in less than 0.5% of autopsied patients. Pulmonary hamartoma is discovered in men more often than in women by a ratio of 2 : 1, with an average age at diagnosis over 60 years old^2^,^3^. Pulmonary cystic hamartoma was first described in 1986, predominantly comprised of immature mesenchymal cells from multiple bilateral cysts and nodules. Until 2012, only 15 cases of this disease were reported in the literature^4^. This report study was composed based on the Surgical CAse REport (SCARE) 2020 guidelines^5^.

## Case presentation

A 43-year-old Indonesian female complained of chest and right shoulder pain, especially in the right clavicle and a mass around her right shoulder that never got bigger, measuring about 2 cm. The pain around the mass did not last long. The patient’s medical history showed that she had pulmonary tuberculosis 25 years ago, was triumphantly treated for 6 months, and was declared cured. She got coronavirus disease 2019 in 2020 one time.

**Figure 1:**
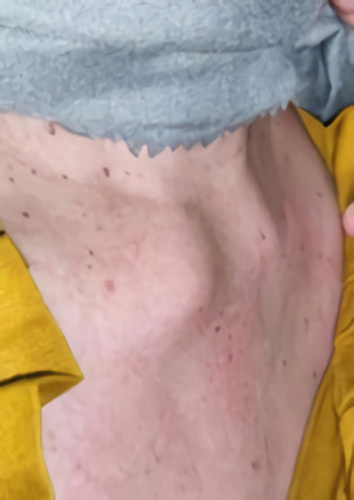
The right clavicula region shows a solid mass.

**Figure 2:**
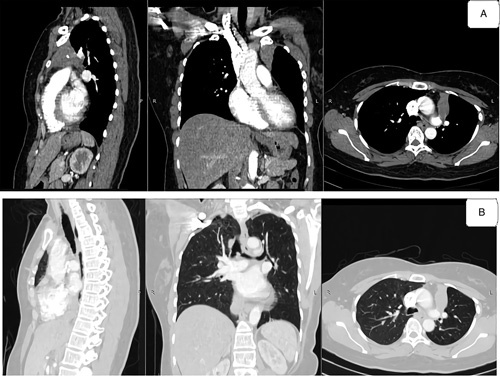
Computed tomography scan thorax contrast.

**Figure 3:**
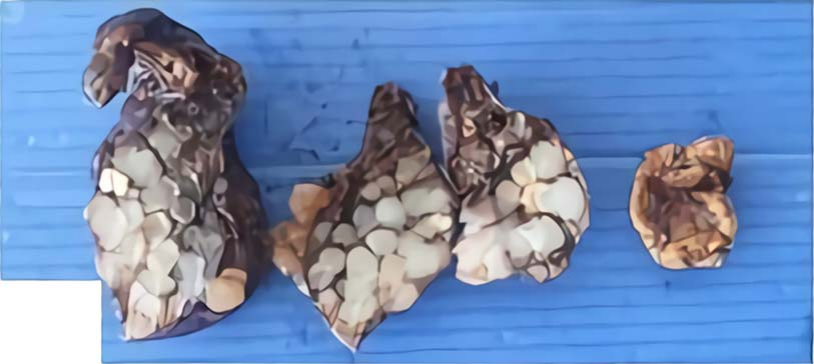
Pathological anatomy laboratory gross of the specimen shows around white and cartilaginous tissue.

Physical examination showed a hard solid mass of about 2 cm (inspection), fixed and without pain (palpation) in the right clavicula region (sternoclavicular joint side; Fig. 1). The patient also had an increase in the jugular vein. Chest X-ray found homogenous opacity in the mediastinum region. Thoracic computed tomography scan with contrast found enhancing solid mass (35 HU) with calcification, regular border, lobulated, measurement about 3.3×2.5×5.9 cm in anterior mediastinal suggesting thymoma (Fig. 2). The tumor marker [lactate dehydrogenase (LDH), alpha-fetoprotein (AFP), and beta-human chorionic gonadotropin (β-HCG)] found no abnormality. This study performed a left thoracotomy excision on the patient, and the mass characteristic was solid and mucinous (Fig. 3).

**Figure 4:**
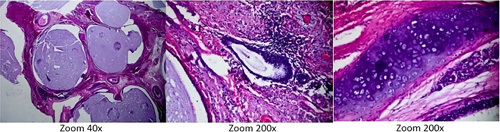
Microscopic showing mostly cartilage, fibroblastic background, bronchial epithelial lining cells, hyaline cartilage, and cysts tissue.

Postsurgery, anatomical pathology results showed the mass with a distinct multicystic area layered by columnar epithelial filled in with myxoid and more, seromucous gland, alveoli tissues, and erythrocytes inflammatory cells (Fig. 4). The conclusion from these microscopic results was pulmonary cystic hamartoma. The primary marker was the absence of a distinct boundary between the mass and lung parenchyma, and there was superior lobe hypoplasia in the left lung. The patient had a good prognosis as she had no problems with daily activities, no swelling, no pain, and no sequelae. A similar condition was also found at a follow-up 3 months later.

## Discussion

Hamartomas are caused by aberrant development of normal tissue and can occur infrequently or as part of a syndrome. Hamartoma is caused by a developmental error and may appear in several sites. It grows at the same rate as the original tissue. Some genes are involved in the pathogenesis of hamartoma development, including *SMAD4*, *PTEN*, *STK1*, and *BMPR1A*. Many hereditary syndromes are associated with hamartoma formation, including tuberous sclerosis, Cowden syndrome, PTEN hamartoma tumor syndrome, Peutz–Jeghers syndrome, and other genetic diseases of hamartoma development^6^,^7^.

Most people with pulmonary hamartoma have no symptoms. However, some may experience respiratory symptoms such as hemoptysis, cough, phlegm, or chest pain^11^. Surgical treatment is used to treat pulmonary hamartoma. The timing and indications for surgical treatment of pulmonary hamartoma are still debatable. Guo *et al*. retrospectively reviewed research of a 20-year clinical history of surgical treatment for 39 patients with pulmonary hamartoma between 1985 and 2006. Wedge resection, enucleation, segmentectomy, lobectomy, and pneumonectomy were among the 40 surgeries performed on these 39 patients. Because pulmonary hamartoma is a slow-growing mass that rarely expands, this research recommends a step-by-step approach when a clinical physician decides on operative treatment. Surgical excision is justified and should be required when a solitary pulmonary lesion measured more than 2.5 cm in diameter or the probability of malignancy cannot be ruled out. In terms of surgery indications, Guo *et al*.^8^ came up with the following summary: (1) a solitary pulmonary lesion with a diameter larger than 2.5 cm; (2) heavy psychic burden making it necessary to remove the lesion; (3) a tendency for expansion or recurrence; (4) pulmonary symptoms unresponsive to drug treatment; and, most importantly, the lesion could not be distinguished from malignancy. The writer’s main choice procedure was wedge resection^9^. Only in the following circumstances was a more aggressive lobectomy or total pneumonectomy considered: the central intrapulmonary hamartoma was located in the deep part of the pulmonary lobes and adhered severely to the hilum of the lung, the distal lung tissue was nonfunctional, and there were multiple or large tumors making wedge resection impossible. The importance of the intraoperative frozen section must be addressed to avoid disregarding any malignant lesions, and normal lung tissue should be saved as much as possible^10^–^12^.

There were 23 males and 15 females in a retrospective study conducted by Erdogu *et al*., and lung cancer and hamartoma were found simultaneously in four cases at the time of diagnosis. Solitary pulmonary nodules with benign radiological findings were typical with pulmonary hamartoma. Although hamartomas can be related to lung cancer at the time of diagnosis or follow-up, it is essential to remember that a different nodule detected in hamartoma patients could be linked to lung cancer^13^. Ekinci *et al*.^14^ found 96 patients with pulmonary hamartomas, with 26 women and 70 men in the group. Malignancies were detected in 23 patients, and their results showed that patients with pulmonary hamartomas might have coexisting lung malignancies.

## Conclusion

Pulmonary hamartoma is a rare case. Management of pulmonary hamartoma is complete resection of the pulmonary hamartoma. Pathological anatomy examination plays a vital role in determining the type of tumor.

## Ethical approval

This case report does not require any ethical approval.

## Patient consent

Written informed consent was obtained from the patient to publish this case report and accompanying images. A copy of the written consent is available for review by the Editor-in-Chief of this journal on request.

## Sources of funding

None.

## Author contribution

All authors contributed to data analysis, drafting and revising the paper, giving final approval of the version to be published, and agreeing to be accountable for all aspects of the work.

## Conflicts of interest disclosure

All authors declare that they have no conflicts of interest.

## Research registration unique identifying number (UIN)

1. Name of the registry: NA.

2. Unique identifying number or registration ID: NA.

3. Hyperlink to your specific registration (must be publicly accessible and will be checked): NA.

## Guarantor

Anna Febriani is the person in charge of the publication of our manuscript.

## Provenance and peer review

Not commissioned, externally peer-reviewed.
